# ROS Detoxification and Proinflammatory Cytokines Are Linked by p38 MAPK Signaling in a Model of Mature Astrocyte Activation

**DOI:** 10.1371/journal.pone.0083049

**Published:** 2013-12-23

**Authors:** Adrian Nahirnyj, Izhar Livne-Bar, Xiaoxin Guo, Jeremy M. Sivak

**Affiliations:** 1 Department of Vision Sciences, Toronto Western Research Institute, University Health Network, Toronto, Ontario, Canada; 2 Department of Ophthalmology and Vision Science, University of Toronto, Toronto, Ontario, Canada; 3 Department of Laboratory Medicine and Pathobiology, University of Toronto, Toronto, Ontario, Canada; Hannover Medical School, Germany

## Abstract

Astrocytes are the most abundant glial cell in the retinal nerve fiber layer (NFL) and optic nerve head (ONH), and perform essential roles in maintaining retinal ganglion cell (RGC) detoxification and homeostasis. Mature astrocytes are relatively quiescent, but rapidly undergo a phenotypic switch in response to insult, characterized by upregulation of intermediate filament proteins, loss of glutamate buffering, secretion of pro-inflammatory cytokines, and increased antioxidant production. These changes result in both positive and negative influences on RGCs. However, the mechanism regulating these responses is still unclear, and pharmacologic strategies to modulate select aspects of this switch have not been thoroughly explored. Here we describe a system for rapid culture of mature astrocytes from the adult rat retina that remain relatively quiescent, but respond robustly when challenged with oxidative damage, a key pathogenic stress associated with inner retinal injury. When primary astrocytes were exposed to reactive oxygen species (ROS) we consistently observed characteristic changes in activation markers, along with increased expression of detoxifying genes, and secretion of proinflammatory cytokines. This *in vitro* model was then used for a pilot chemical screen to target specific aspects of this switch. Increased activity of p38α and β Mitogen Activated Protein Kinases (MAPKs) were identified as a necessary signal regulating expression of MnSOD, and heme oxygenase 1 (HO-1), with consequent changes in ROS-mediated injury. Additionally, multiplex cytokine profiling detected p38 MAPK-dependent secretion of IL-6, MCP-1, and MIP-2α, which are proinflammatory signals recently implicated in damage to the inner retina. These data provide a mechanism to link increased oxidative stress to proinflammatory signaling by astrocytes, and establish this assay as a useful model to further dissect factors regulating the reactive switch.

## Introduction

Astrocytes play a critical role in maintaining neuronal homeostasis in the central nervous system (CNS) through secretion of trophic factors, neurotransmitter recycling, nutrient and oxygen balancing, and free radical scavenging [1]–[4]. In response to injury or stress, astrocytes undergo a phenotypic switch, characterized by; upregulation of intermediate filament proteins, such as glial fibrillary acidic protein (GFAP) and vimentin, loss of glutamate buffering function, secretion of pro-inflammatory cytokines, and increased production of antioxidants [2], [4], [5]. Both positive and negative influences of astrocyte re-activation have been implicated in a wide variety of neurodegenerative processes. However, the intracellular mechanism regulating this switch remains poorly understood due, in part, to a need for responsive models of mature cells [6]. Consequently, pharmacologic strategies to modulate selective aspects of this process have not been thoroughly explored.

As an embryonic outpocketing of the forebrain, the retina is a common model for CNS damage, due in part to its metabolic sensitivity, and environmental exposure. Accumulated oxidative damage has been implicated as a central pathogenic stress associated with common diseases of the aging retina, such as diabetic retinopathy and glaucoma [7]–[10]. In the adult eye, astrocytes migrate out of the optic nerve head (ONH) into the retinal nerve fiber layer (NFL) by P9, and assume a quiescent phenotype by weaning [11]. Along with astrocytic radial Müller glia, they rapidly re-activate following oxidative stress [4], [12] and have been proposed to both preserve inner retinal tissue homeostasis, and generate a detrimental para-inflammatory response [13]–[16]. These effects are accomplished through increased antioxidant activity, and secretion of proinflammatory cytokines that activate resident microglia, increase vascular permeability, and induce direct damage or protection to retinal ganglion cells (RGCs) [4], [14], [17], [18]. However, the molecular link between oxidative stress and proinflammatory signaling in these cells has not been established.

Here we describe a system for rapid isolation and culture of mature astrocytes from the adult rat retina that remain relatively quiescent, but can be induced to respond robustly when challenged with titrated levels of reactive oxygen species (ROS). A pilot chemical screen identified p38α and β mitogen activated protein kinase (MAPK) activity as a key signal regulating specific components of this switch. The p38 MAPKs are serine-threonine kinases mediating responses to environmental stress in the CNS. Inhibition of p38 signaling was assessed against expression of antioxidant genes, ROS mediated cell death, and multiplex profiling of key growth factors and proinflammatory cytokines implicated in damage to the inner retina. These data provide a mechanism to link increased oxidative stress to proinflammatory signaling by astrocytes, and establish this assay as a useful model to further dissect factors regulating the reactive switch.

## Materials and Methods

### Primary Adult Retinal Astrocyte Isolation and Culture

Mature retinal astrocytes were isolated and cultured from adult Wistar rats by adapting a protocol that is well established for neonatal astrocyte brain cultures [19]–[22], with modifications to incorporate media and methods we have used to culture mature human ONH astrocytes [17]. Briefly, weaned 21–28 day old rats were sacrificed by CO_2_ asphyxiation in accordance with a protocol approved by the animal care and use committee of the University Health Network. Eyes were rapidly enucleated and immediately placed in ice cold MEM-H17 supplemented with 5% fetal bovine serum, 5% horse serum and 1% penicillin/streptomycin. Retinas were dissected and digested on an orbital shaker for 20 minutes in serum-free MEM-H17 supplemented with 1.5 mg/ml trypsin (Sigma, St. Louis, MO) and 5****µg/ml DNase 1 (Roche). After washing, cells were mechanically dissociated by trituration, and centrifuged to separate viable cells from debris, before being seeded into polystyrene tissue culture dishes. To enhance attachment and growth of primary astrocytes during the first 24 h, cells were cultured in specialized astrocyte growth media (Lonza, Basel, CH), which was subsequently replaced with complete MEM-H17 media. Cells were grown to confluence (8–9 d) at which point they were placed on an orbital shaker for 6 h at 110 rpm. For neonatal brain cultures, orbital shaking is used to remove oligodendrocytes and microglia [20] and we employed the same technique to isolate mature retinal cultures. The resulting cells were characterized by morphology and immunofluorescence staining, as described in the results. In all experiments presented n refers to the number of independent cultures analyzed.

### Ethics Statement

All animal work was performed under a protocol approved by the Animal Care Committee of the University Health Network, in accordance with all relevant national and international guidelines.

### Oxidative Stress Model

Cells were exposed to H_2_O_2_ or paraquat (Sigma) at the indicated concentrations and for the indicated time. SB203580 (Selleckchem, Houston, TX) was dissolved in DMSO and added to culture media at the indicated final concentration 1 h before application of stress. For analyses of ROS, cells were gently detached with TrypLE and collected in suspension, then incubated with DCF (5-(and-6)-chloromethyl-2′,7′-dichlorodihydrofluorescein diacetate, Invitrogen), dissolved in DMSO, and added to culture media at a final concentration of 4 µM for 45 min, followed by a wash and incubation for 10 min in fresh media. Cell death was measured by propidium iodide (PI) (Sigma), dissolved in H_2_O and diluted to a working solution in PBS of 10 mg/ml. Collected cells were analyzed using a FACSCalibur flow cytometer (BD Biosciences, Franklin Lakes, NJ) at 530 nm (DCF), and 670 nm (PI). All FACS experiments were done in triplicate, with >10,000 cells per well, from at least three independent cultures.

### Immunofluorescence Microscopy

Cells were washed, fixed in 4% PFA for 15 min, permeabilized in 0.1% Triton-X 100 for 20 min, and blocked with 5% BSA for 1 h. Cells were probed with primary antibodies to: GFAP-cy3 (Sigma), Vimentin (Sigma), S100A (Cedarlane), Glutamine Synthetase (GS) (Abcam), Pax-2 (Covance), CD68 (Biolegend), Brn-3 (Santa Cruz Biotechnology Inc.), CD31 (BD Pharmingen). Primary antibodies were incubated O/N at 4°C, followed by the appropriate Alexa Fluor 488 nm or 568 nm secondary antibodies (Life Technologies, Burlington, ON), and mounted with VectaShield anti-fade medium with DAPI (Vector Labs, Burlingame, CA). Imaging was performed with a Nikon TIE2 inverted microscope. For tissue sections, eyes were dissected from adult Wistar rats and fixed in 4% paraformaldehyde (PFA), and equilibrated in 30% sucrose overnight, embedded in OCT, and stored at −80°C until sectioning. Cryosections were mounted on SuperfrostPlus slides (VWR, Radnor, PA), and stored at −80°C. For immunofluorescence staining sections were dried at room temperature (RT) and fixed in 4% PFA for 10 min, and rinsed in PBS-T. Blocking was performed with 5% serum from 2° antibody host, in a humidified chamber for 1 h at RT, followed by primary antibody for >1 h at RT or at 4° overnight, washed, and incubated with secondary antibody for 1 h, RT, mounted and imaged as above.

### RNA Analysis

Total RNA was isolated from cells samples using TRIZOL reagent (Life Technologies), and treated with RQ1 DNase (Promega), as per the manufacturer’s instructions. cDNA was synthesized using Superscript III Reverse Transcriptase according to the manufacturer’s instructions (Life Technologies). Forward and reverse primers were designed using Primer3 0.4.0 (http://primer3.wi.mit.edu). Primer sequences are presented in Table 1. One µl of each cDNA sample was added to a master mix containing RNase-free water, Power SYBR Green (Applied Biosystems, Carlsbad, CA) and primer mix (200 nM final, Sigma) in a 10 µl reaction volume. Real-time PCR was performed using an Eppendorf Realplex2. A melting curve was constructed in order to ensure optimal primer binding and efficiency of cDNA amplification. Relative abundance of target mRNA was determined from Ct values and normalized to the geometric mean of the housekeeping gene, TATA binding protein (TBP). Results were analyzed by one-way ANOVA using Tukey’s multiple comparison test.

**Table 1:** RT-qPCR primer sequences.

Gene	Forward	Reverse
Catalase	5′-AAGCTGGTTAATGCGAATGG-3′	5′-CAAGTTTTTGATGCCCTGGT-3′
Gpx-1	5′-TGAGAAGTGCGAGGTGAATG-3′	5′-CGGGGACCAAATGATGTACT-3′
HO-1	5′-TCTATCGTGCTCGCATGAAC-3′	5′-AAGGCGGTCTTAGCCTCTTC-3′
MnSOD	5′-GGCCAAGGGAGATGTTACAA- 3′	5′-GCTTGATAGCCTCCAGCAAC- 3′
TBP	5′-ACAGGTGGCAGCATGAAGTG-3′	5′- GCAGGGTGATTTCAGTGCAGA-3′

### Protein Analysis

For western blots, cells were harvested in TrypLE (Invitrogen) and washed in PBS, before being, lysed in RIPA buffer (Cell Signaling), augmented with Complete Mini EDTA-free protease inhibitor (Roche), and PhosSTOP phosphatase inhibitor (Roche). Total protein was quantified and equal concentrations were submitted to SDS-PAGE by standard methods. Proteins were transferred to PVDF membrane and probed with antibodies raised against GFAP (Sigma), HSP70 (Santa Cruz), PGC-1α (Novus), GS (Abcam), ph-p38 and pan p38 MAPK (Cell Signaling), and GAPDH (Calbiochem), and detected with appropriate IRDye secondary antibody (Li-Cor Biosciences, Lincoln, NE). Blots were imaged and analyzed for intensity with an Odyssey infrared imaging system (Li-Cor Biosciences), with each band being normalized to a GAPDH loading control. Results were analyzed by one-way ANOVA using Dunnet’s multiple comparison test. Multiplex cytokine analyses of culture media was performed by bead technology, which compared measured values for each sample to a standard curve for each analayte (EVE Technologies, Calgary, AB). An ELISA kit for BDNF was supplied by Millipore. Briefly; conditioned media was added to a 96 well plate pre-coated with mouse anti-human/rat BDNF monoclonal antibody. Bound BDNF was then incubated in a BDNF specific biotin conjugated antibody, followed by streptavidin-enzyme, substrate, and stop solution. The plate was read at 450 nm. A standard curve was generated, establishing a relationship between optical density and BDNF concentration, which allowed for accurate quantification of sample BDNF concentrations. Results were analyzed by one-way ANOVA using Tukey’s multiple comparison test.

## Results

### Culture and Characterization of Mature Retinal Astrocytes

**Figure 1:**
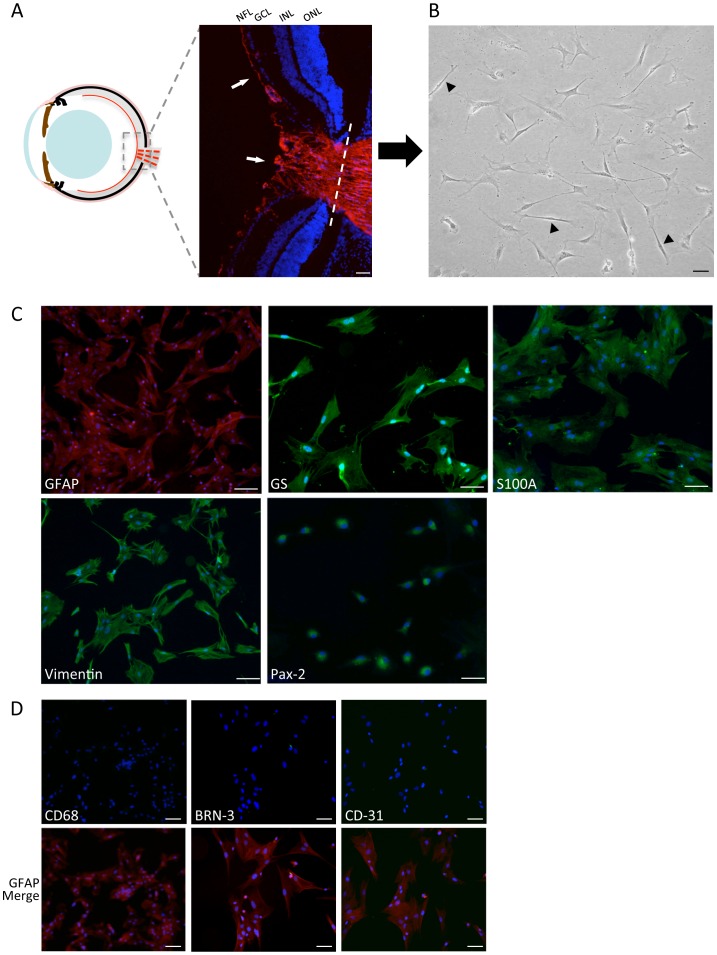
Culture and characterization of mature primary retinal astrocytes. **A)** A cartoon cross-section shows the restricted location of astrocytes in the adult eye (red lines). The inset fluorescent image corresponds to the ONH region stained for GFAP (red, arrows), with the dotted line indicating the site of detachment. **B)** Rapid dissociation and selective mature astrocyte culture conditions produced stellate, multi-processed cells that gradually transitioned to a polygonal morphology. A minority of cells displayed a distinct bipolar appearance characteristic of Müller glia (arrowheads). **C)** Cells were stained positively for astrocyte markers GFAP, vimentin, GS, Pax-2, and S100A. **D)** Cells did not stain for CD68, BRN3, or CD31 (green channel), but remained positive for GFAP (corresponding red channel merge below each panel). Nuclei are stained with DAPI (blue). (Scale bar = 50 µM, NFL; nerve fiber layer, GCL; ganglion cell layer, INL; inner nuclear layer, ONL; outer nuclear layer).

Mature astrocytes were isolated and cultured as described from weaned rat retinas, and characterized by morphology and immunofluorescence staining for a panel of astrocytic markers and potential contaminating cell types. Under optimized conditions, cultures were consistently ≥98% positive for astrocyte proteins GFAP, Vimentin, GS, Pax-2, and S100A, as we have previously reported as markers of human ONH astrocytes [17] (Figure 1C). Cultures were negative for the microglial marker CD68, RGC maker BRN3, and vascular endothelial cell marker CD-31 (Figure 1D).

The cells exhibited stellate processes that gradually transitioned to a polygonal morphology typical of monolayer astrocyte cultures (Figure 1B). However, in each culture we observed a small number of distinct bipolar cells with the appearance of Müller glia (Figure 1B). Müller cells are specialized glia with astrocytic properties that span the neuroretinal layers and retain a radial morphology [23]. Mature Müller cells are normally negative for many astrocyte markers, but can induce their expression when activated, and visa versa [23]–[27]. However, to our knowledge, Pax-2 has only been detected in astrocytes and not Müller glia in the rodent eye [11], [28], [29]. We confirmed that nuclear Pax-2 immunoreactivity was present in the NFL that co-localized with GFAP containing cells, but was not present in the inner nuclear layer, or any other retinal compartment (Figure S1). When stained with the same antibody our primary cultures were highly Pax-2 positive in nearly all cells, regardless of morphology (Figure 1C). These results increase our confidence that our mature cultures are highly enriched in NFL astrocytes. However, out of caution the percentage of cells exhibiting Müller-like morphology in our primary cultures were scored by two observers from replicate images of three independent cultures. On average 5% (±2.1) had Müller-like morphology, 87% (±3.9) were astrocytic, and 8% (±5.9) were scored as unclear. Therefore, these populations appear to be mature astrocytic cultures that are highly enriched for NFL and ONH astrocytes.

### Oxidative Stress Induces a Sensitive and Robust Reactive Response in Freshly Cultured Astrocytes

**Figure 2:**
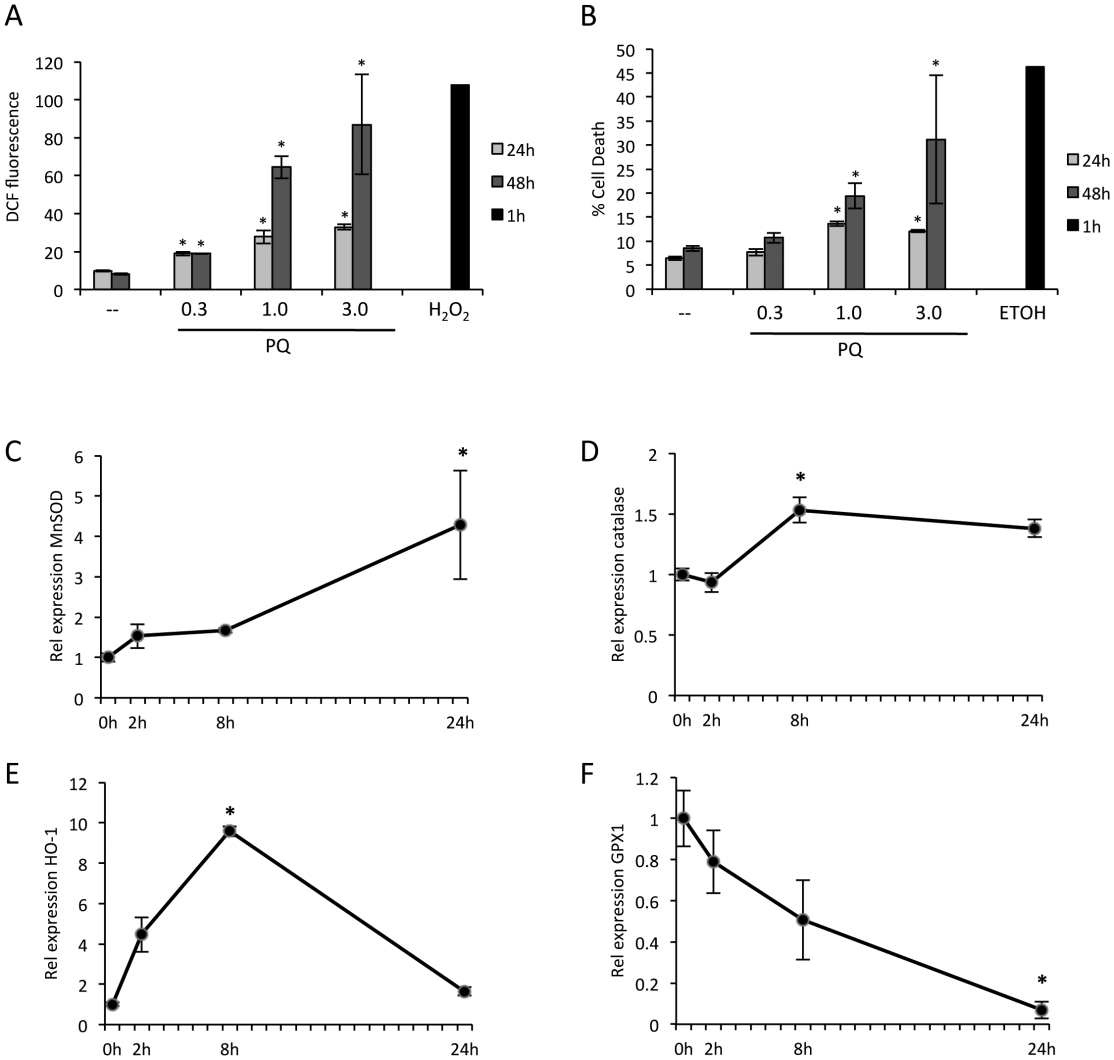
ROS exposure in primary mature astrocytes induces robust dose and time-dependent cell death and detoxifying responses. **A)** Exposure of primary astrocytes to increasing mM concentrations of PQ results in dose and time dependent ROS (DCF fluorescence) over 24 and 48 hours, as measured by flow cytometry. This result can be compared to acute exposure to 0.5 mM H_2_O_2_, which generates a sharp spike in peroxide formation by one hour (n = 3, bars represent S.E., *p<0.05). **B)** Dose and time dependent increases in cell death (PI signal), at 24 and 48 hours, following exposure to increasing mM concentrations of PQ. ETOH was used as a positive control for cell death after 1 hour (n = 3, bars represent S.E.). **C–F)** Time course of RT-qPCR results from cells exposed to 1 mM PQ shows significant increases in expression of MnSOD, catalase, HO-1, and GPX1 by eight hours (n = 4, *p<0.05 at peak/trough, bars represent S.E.).

To generate oxidative stress, astrocytes were exposed to acute damage by H_2_O_2_, or to the redox cycling compound, paraquat (PQ). PQ is a common herbicide that regenerates itself through production of superoxide radicals, resulting in chronic neurotoxic damage. PQ has become an established tool for generating a consistent level of oxidative stress *in vitro* [30], [31]. An advantage of this method is the application of a slower, more sensitive stress than H_2_O_2_. For initial experiments increasing concentrations of PQ were tested using flow cytometry to identify an exposure that generated increased ROS and subsequent cell death that were measurable, but not immediately cytotoxic. Concentrations of 0.3, 1.0, and 3.0 mM PQ generated dose-dependent increases in ROS over 24 hours that were exacerbated at 48 hours. In contrast, 500 µM H_2_O_2_ generated a sharp increase in ROS by 1 hour (Figure 2A). The same concentrations of PQ induced corresponding dose dependent increases in cell death (Figure 2B). In this case 70% ETOH was used as a positive control for cell death. Based on these data, PQ concentrations of 0.3 and 1.0 mM were used for further experiments.

To address the molecular responses of cell cultures to ROS, a series of stress and detoxifying genes were assessed by quantitative real-time RT-PCR (RT-qPCR) over a 24-hour time course following exposure to 1.0 mM PQ. Significant increases were observed for the detoxifying genes heme oxygenase (HO-1), catalase, and manganese superoxide dismutase (MnSOD) (Figure 2C–E), and a decrease for glutathione peroxidase (GPX1) (Figure 2F). Based on these results an eight-hour time point was chosen for assessment in further RT-qPCR experiments.

**Figure 3:**
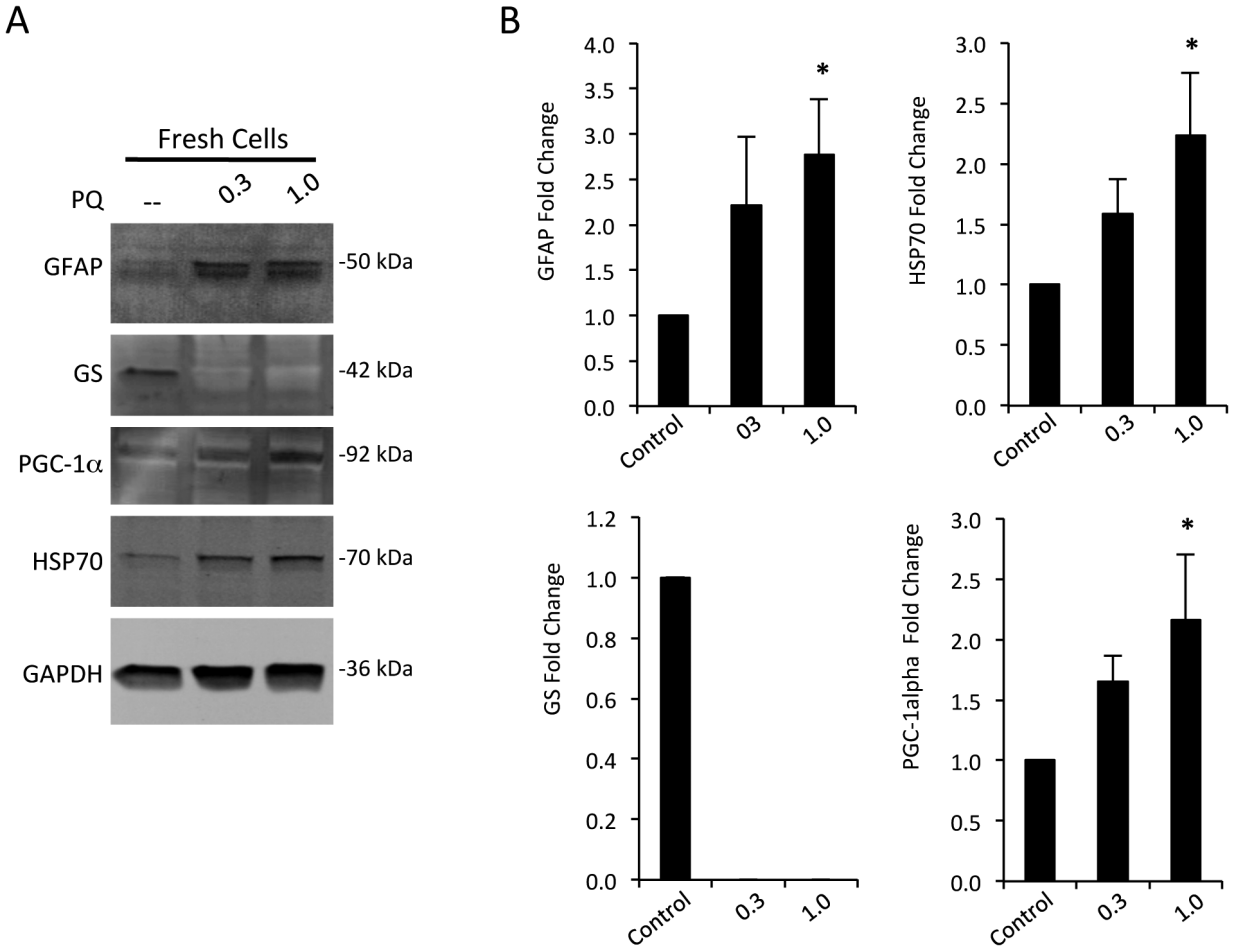
Oxidative stress mediates robust induction of activation and stress markers in mature retinal astrocytes. **A)** Representative western blots from cells exposed to 0.3 and 1.0 mM PQ, and control, for 24 hours. Dose dependent increases were observed for GFAP, HSP70, PGC-1α, and there was a dramatic decrease in GS, compared to a loading control GAPDH. **B)** Corresponding quantification of repeated experiments as in (A) showing consistent responses for each protein probed from fresh cultures (n≥6 independent cultures, *p<0.05, bars represent S.E.).

To assess the biochemical response of the cells under these conditions, a series of western blots were probed for established markers of glial reactivity and of oxidative and metabolic stress. In freshly cultured cells (three passages or less), concentrations of GFAP, the heat shock protein HSP70, and metabolic and oxidative stress regulator PGC-1α (peroxisome proliferator-activated receptor γ co-activator-1α), increased progressively in response to 0.3 and 1.0 mM PQ over 24 hours. In contrast, the level of the key glutamate recycling enzyme, glutamine synthetase (GS), was dramatically decreased (Figure 3A–B). Increased GFAP and decreased GS are considered hallmarks of astrocyte reactivity [32], [33], but are often difficult to reproduce *in vitro*. Increases in HSP70 and PGC-1α have been previously noted in astrocytes in response to oxidative stress [21], [34]. In comparison, subcultured cells (passaged ≥ four times) often showed reduced, or absent responses; no increased GFAP, or PGC-1α was detected, GS was not present in untreated cells, and basal concentrations of HSP70 were elevated (Figure S2). For this reason, all subsequent experiments conservatively used cells that were passaged ≤3 times.

### Astrocyte Activation Induced by Oxidative Stress Depends on p38 MAPK Signaling

**Figure 4:**
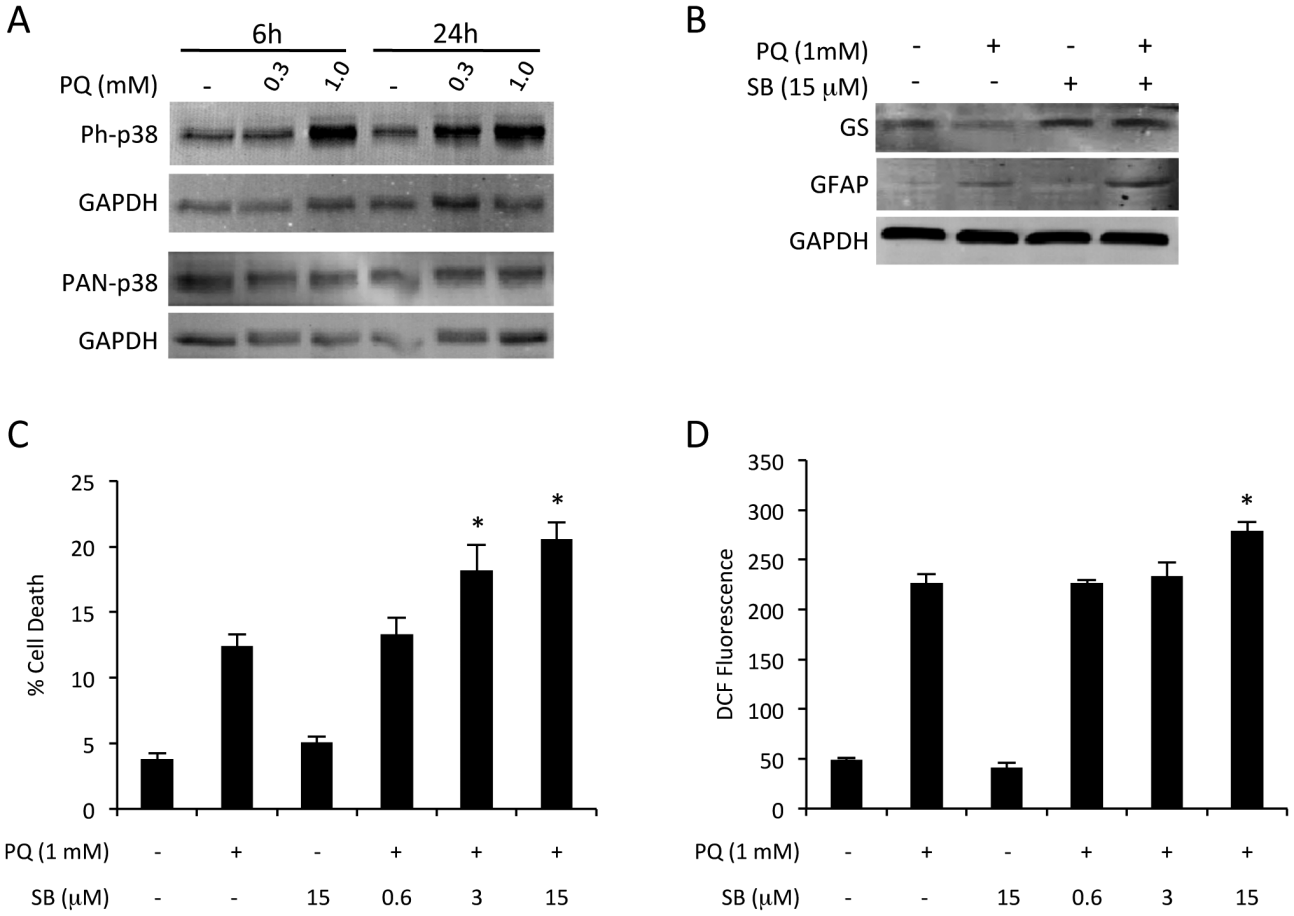
Oxidative stress induces p38 MAPK dependent astrocyte responses. **A)** Western blotting showed increased phosphorylation of p38 MAPK induced by escalating concentrations of PQ at 6 and 24 hours. **B)** Application of 15 µM SB for 24 hours blocked oxidative stress induced loss of GS, but had little effect on GFAP induction. **(C–D)** Exposure to increasing concentrations of SB in the presence of oxidative challenge significantly increased (C) cell death, and (D) ROS, in a dose dependent manner (n = 3 cultures, *p<0.05 compared to PQ alone, bars represent S.E.).

Since oxidative stress induced such robust responses, we used this model to begin dissecting the regulatory cascades involved. In this regard, PQ induced oxidative stress forms an attractive assay for pharmacologic screening, as it is rapid, quantitative, and scalable. Also, both the degree of stress (ROS), and result (cell death), can be simultaneously measured. In this manner, we performed a pilot screen with a panel of small molecule inhibitors measuring changes to both cell death and ROS. The compound SB203580 (SB) is a potent and specific inhibitor of p38 α and β MAPK isoforms [35]. Strongly increased p38 MAPK phosphorylation was confirmed following ROS exposure after 6 and 24 hours (Figure 4A). Addition of 15 µM SB blocked PQ-induced loss of GS, but had small effect on GFAP induction (Figure 4B). Increasing concentrations of SB in combination with oxidative stress resulted in dose dependent increases in both ROS and cell death (Figure 4C–D). Application of SB alone had no effect (Figure 4C–D).

**Figure 5:**
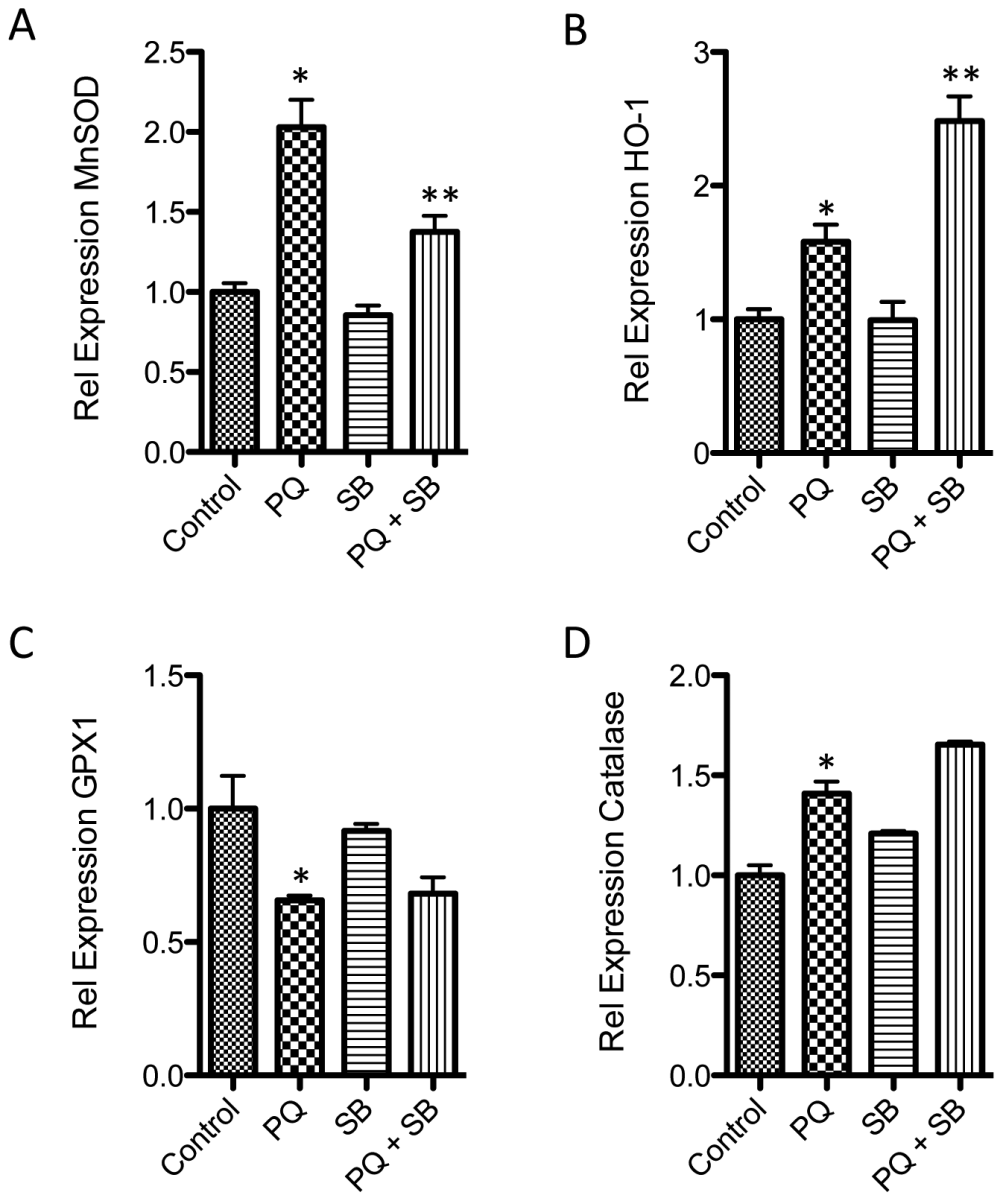
p38 MAPK modulates expression of specific stress and detoxifying genes. RT-qPCR of cDNAs from cells exposed to oxidative stress (1 mM PQ), in the presence or absence of SB. Inhibition of p38 MAPK activity with 15 µM SB (**A**) blocked increased expression of MnSOD and (**B**) exasperated expression of HO-1. No significant p38 dependent changes were observed for Gpx1 or catalase (*p<0.05 compared to control, **p<0.05 compared to PQ, n = 4, bars represent S.E.).

To determine the mechanism underlying this effect on ROS, the influence of p38α and β MAPK inhibition on expression of antioxidant gene expression was evaluated by RT-qPCR. Based on the prior time courses (Figure 2), an eight-hour point was chosen for analyses of cDNA from cells exposed to PQ, SB, PQ+SB, or control. Application of 15 µM SB significantly blocked the oxidative stress induced increase in MnSOD, and enhanced the increase in HO-1 (Figure 5A–B). No changes were observed between control and SB treatment alone, suggesting the effects observed are a specific reaction to the application of oxidative stress. There was little or no effect of SB on GPX1 or catalase expression (Figure 5C–D).

### Cytokine Profiling Identifies p38 MAPK Mediated Secretion of IL-6, MIP-2, and MCP-1

**Figure 6:**
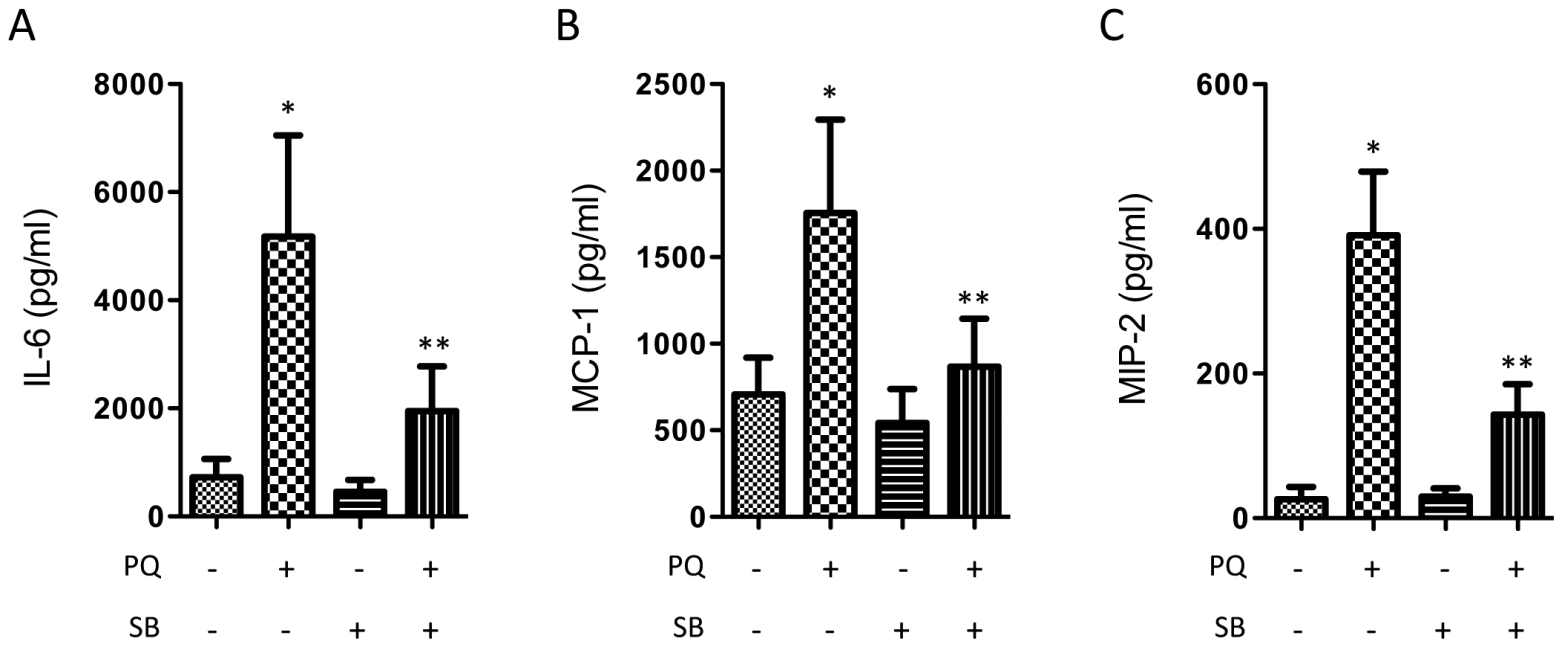
p38 MAPK regulates secretion of specific proinflammatory cytokines. Conditioned media were collected from control primary astrocytes, and from cells treated with 1+SB for 24 hours. Significant increases were observed for **A)** IL-6, **B)** MCP-1, and **C)** MIP-2, which were blocked by p38 MAPK inhibition (n = 6, *p<0.05 compared to control, **p<0.05 compared to PQ alone, bars represent S.E.).

Astrocyte conditioned media were analyzed by multiplex cytokine profiling in order to determine the effect of oxidative stress and p38 MAPK signaling on key secreted factors that might signal to surrounding neurons, microglia, vascular, and inflammatory cells. Freshly isolated astrocytes were subjected to PQ, SB, PQ+SB, and control conditions, for 24 hours, as described above. Conditioned media was collected and submitted to multiplex and ELISA analyses for a panel of 29 key cytokines and growth factors (Table 2). Of the analytes, 12 were below levels of quantitation (generally 1 pg/ml), and a further 8 showed no significant change in response to oxidative stress, including IL-1β, TNFα, and TGF- β. Concentrations of GRO/KC, IL-18, MIP-1α, VEGF, and IL-10 were significantly increased by oxidative stress, but this change was not p38 MAPK dependent (Table 2). Only the proinflammatory cytokines IL-6, MIP-2, and MCP-1, showed significant p38 MAPK-dependent increases compared to control and oxidative stress groups (Figure 6).

**Table 2:** Relative protein concentrations in conditioned media.

Analyte	Control	PQ	SB	PQ & SB
GRO/KC	1.00	8.53*	2.15	7.36
**MIP-2**	**1.00**	**8.03** *	**1.05**	**3.87** **
IL-18	1.00	6.01*	1.21	8.45
LIX	1.00	4.81	1.57	3.90
**IL-6**	**1.00**	**4.77** *	**0.63**	**1.80** **
MIP-1α	1.00	3.15*	3.26	3.08
**MCP-1**	**1.00**	**2.48** *	**0.77**	**1.23** **
VEGF	1.00	1.97*	0.98	1.55
IL-13	1.00	1.94	0.77	1.27
IL-10	1.00	1.93*	1.05	1.61
RANTES	1.00	1.75	1.68	1.25
IL-4	1.00	1.57	1.12	1.13
IP-10	1.00	1.38	1.08	1.23
IL-1α	1.00	1.34	1.02	1.28
TGF-β	1.00	1.06	1.02	0.97
BDNF	1.00	0.74*	0.46	0.99
IL-12p70	1.00	0.74	0.46	0.99
G-CSF	BLQ	BLQ	BLQ	BLQ
Eotaxin	BLQ	BLQ	BLQ	BLQ
GM-CSF	BLQ	BLQ	BLQ	BLQ
Leptin	BLQ	BLQ	BLQ	BLQ
IL-1β	BLQ	BLQ	BLQ	BLQ
IL-2	BLQ	BLQ	BLQ	BLQ
EGF	BLQ	BLQ	BLQ	BLQ
IFNγ	BLQ	BLQ	BLQ	BLQ
IL-5	BLQ	BLQ	BLQ	BLQ
IL-17A	BLQ	BLQ	BLQ	BLQ
Fractalkine	BLQ	BLQ	BLQ	BLQ
TNFα	BLQ	BLQ	BLQ	BLQ

BLQ; below quantification,

* p<0.05 compared to control,

** p<0.05 compared to PQ (n = 6).

## Discussion

Sensitive glial responses have been associated with both positive and negative effects on the CNS, particularly the inner retina and ONH. However, the mechanisms regulating of these responses remain unclear, and pharmacologic methods for selectively targeting them are relatively unexplored. Changes in GFAP expression have been recently linked to NFκB and STAT signaling [36], as well as activation of the LIM homeodomain transcription factor Lhx2 [37]. Here we describe a system for rapid and consistent isolation of mature astrocytes from the adult rat retina, which can be induced to robustly respond to oxidative stress in a p38 MAPK-dependent manner.

### A Model of Mature Astrocyte Reactivity

Critically, these cells appear to maintain a quiescent phenotype for the first three weeks of culture, but during this period are capable of robust responses to titrated levels of oxidative stress, including characteristic changes in markers associated with activation, such as increases in GFAP, HSP70, and PGC-1α, and a dramatic loss of GS, as well as increased expression of ROS detoxifying genes, and cytokine secretion. Loss of GS is a common marker of astrocyte reactivity, and results in reduced glutamate buffering capacity that generates neurotoxic stress [33], [38]–[40], but has been difficult to reproduce *in vitro*. Interestingly, GS has been reported to *increase* in activated Müller cells [25], [26]. Specific loss of GS in NFL and ONH astrocytes could have localized toxic effects on neighboring RGCs that may explain conflicting reports regarding total glutamate levels in glaucomatous eyes [7], [41]. Increased HSP70 has been previously reported in astrocytes in response to oxidative stress, and PGC-1α is induced in the retina in response to metabolic stress, and we have reported it to be increased in astrocytes following oxidative stress [21], [34], [42]. Strikingly, cells subcultured for several weeks completely lost these responses. This system can now be expanded to study astrocyte responses to additional pathologically relevant stresses, such as hypoxia, and biomechanical strain [43], [44].

### p38α and β MAPK Dependent Detoxification and Proinflammatory Responses

This primary culture model was used to identify p38α and β MAPK activity as a key signal mediating components of this switch. The p38 MAPKs are a family of serine threonine kinases that are key signaling intermediates in the CNS response to environmental stress. When activated, they phosphorylate overlapping networks of downstream effectors to regulate cell differentiation, cell death, and cytokine secretion [45]–[47]. The p38 MAPK family includes four main isoforms, designated; α, β, γ, and δ. Of these, the homologous p38α and β isoforms have been associated with CNS degeneration following ischemia [46], [48], RGC injury [49], and in neonatal brain astrocyte apoptosis and oxidative stress [50]–[52].

We found that signaling through p38 MAPK in mature retinal astrocytes regulates loss of GS, coordinates expression of genes maintaining local oxidative balance, and directs secretion of select proinflammatory cytokines implicated in retinal parainflammatory responses. Inhibition of p38α and β significantly increased ROS and corresponding cell death, reduced expression of MnSOD, and increased expression of HO-1. MnSOD is a key enzyme protecting mitochondrial components from oxidative damage by catalyzing detoxification of superoxide anions. Therefore, reduced MnSOD may explain the ROS increases observed with p38 inhibition. In contrast, HO-1 is an enzyme necessary for the catabolism of heme, with the resulting release of free iron radicals [53]. Therefore increased MnSOD, and reduced HO-1 may have similar antioxidant effects *in vivo*.

Out of a panel of 29 common cytokines, only IL-6, MCP-1, and MIP-2 showed p38-dependent increases. IL-6 is a gp130 activating cytokine that can cross the blood brain barrier to have both positive and negative influences in the CNS [54]. In the retina, IL-6 is rapidly induced in various injury models, and has regenerative effects on RGCs [55], [56]. Consistent with our results, Chidlow *et al* also reported strong induction of IL-6, but not IL-1β or TNF-α in an experimental model of glaucoma *in vivo* [55]. We did not observe strong p38 mediated TGF-β secretion, as reported by Yu et al [57], although there was a trend in this direction. However, we did identify MCP-1 and MIP-2 as two additional p38-dependent chemokines. These proinflammatory proteins are primarily associated with recruitment of macrophages and neutrophils, respectively. In the brain, MCP-1 and MIP2 have also been associated with microglial chemotaxis and reactivity and control of blood brain barrier permeability [58], [59]. Dramatically increased levels of both chemokines have been reported in a variety of inner retinal diseases and models [60]–[64], although their precise roles are still unclear.

In conclusion, we have developed a system to study the mechanisms regulating activation of mature retinal astrocytes, and used it to identify a p38 MAPK dependent link between increased oxidative stress and proinflammatory signaling, with potential for neuroprotective and neurotoxic influences. Increased RGC survival is supported through ROS detoxification and IL-6 secretion, but detrimental autocrine and paracrine stimulation of astrocyte and microglial activity may be induced by MCP-1 and MIP-2, as well as locally increased glutamate levels from loss of GS. This model can now be used to dissect p38 signaling to further isolate these responses through specific inhibition of downstream kinase targets, such as MK2 and MSK1/2, or transcriptional regulators like PGC-1α [45], [65]. A better understanding of this mechanism would enable development of new therapeutic strategies to specifically modulate the reactive switch.

## Supporting Information

Figure S1 **Pax-2 stains retinal NFL astrocytes.** Immunofluorescence microscopy of a rat retina stained with antibodies to **A)** Pax-2 (green), and **B)** GFAP (red), only labels astrocyte nuclei and not Müller glia. Panel **C)** shows a merged image. Nuclei are stained with DAPI (blue).
(TIF)

Figure S2 **Subcultured astrocytes become less responsive to oxidative stress.** **A)** Western blotting of whole cell lysates from fresh cultures (passaged up to three times) had consistent increases in GFAP, PGC-1α and HSP70, and a decrease in GS, in response to increasing concentrations of PQ (as described in Figure 3). **B)** Cells that had been subcultured (passaged more than three times) tended to lose their responsiveness to PQ, such that there was little change in the same marker panel. Therefore fresh cultures were used for the subsequent experiments in this report.
(TIF)
