# Supplementary figures and images for: ROS Detoxification and Proinflammatory Cytokines Are Linked by p38 MAPK Signaling in a Model of Mature Astrocyte Activation
Source: PLoS One. 2013 Dec 23;8(12):e83049. doi: 10.1371/journal.pone.0083049 (PMC3871647; doi:10.1371/journal.pone.0083049)

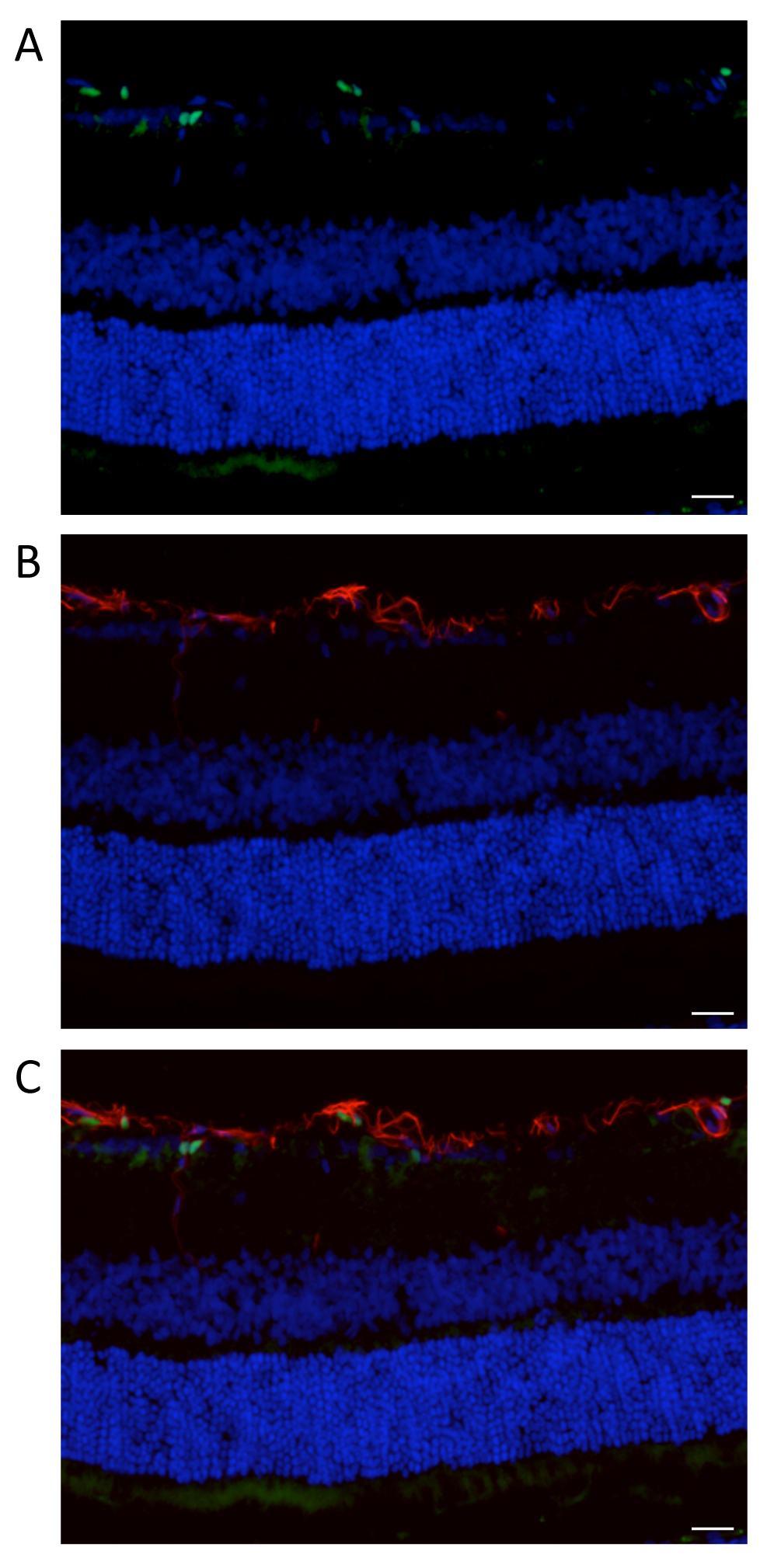

Supplement: Figure S1 — Pax-2 stains retinal NFL astrocytes. Immunofluorescence microscopy of a rat retina stained with antibodies to A) Pax-2 (green), and B) GFAP (red), only labels astrocyte nuclei and not Müller glia. Panel C) shows a merged image. Nuclei are stained with DAPI (blue). (TIF) [file pone.0083049.s001.tif]

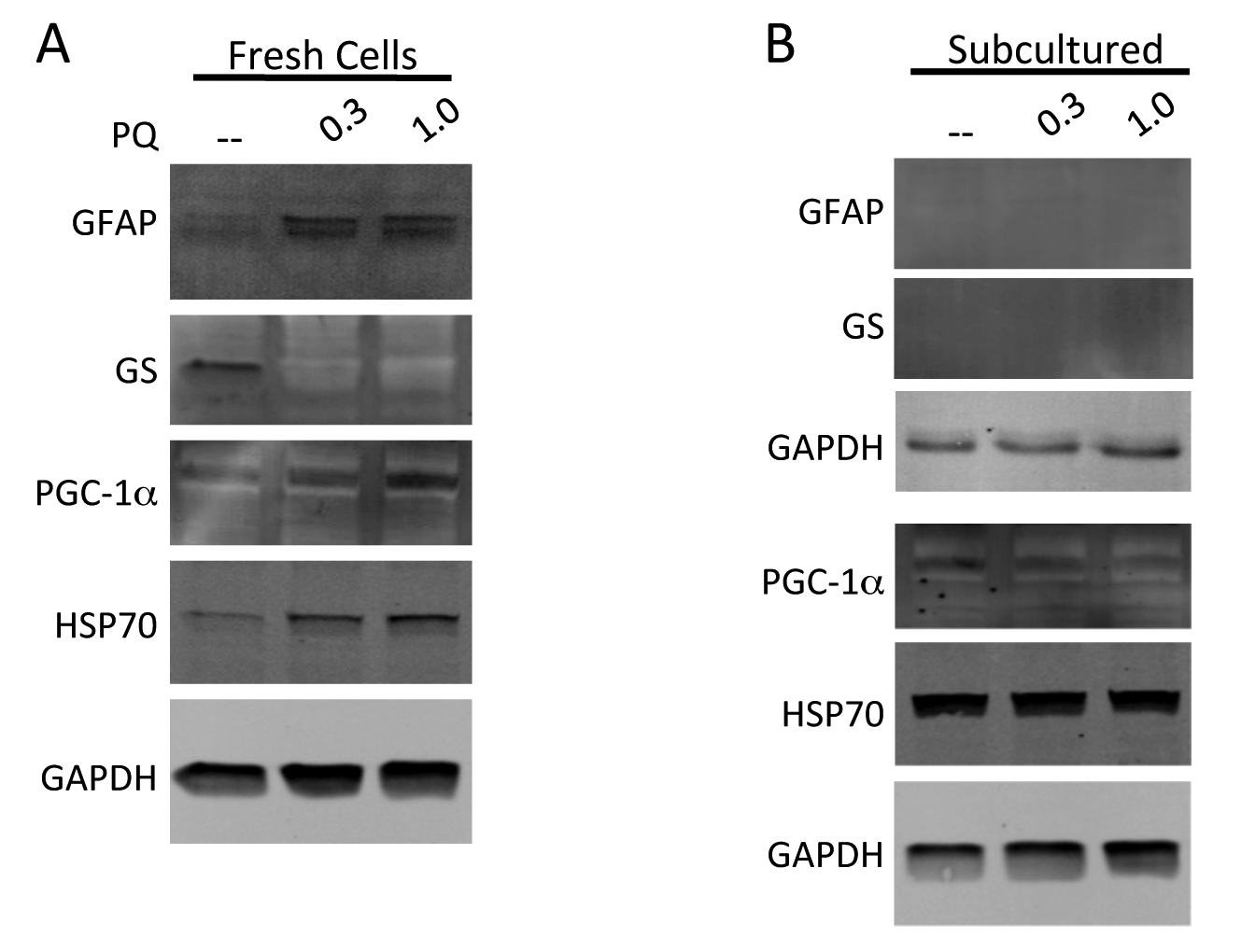

Supplement: Figure S2 — Subcultured astrocytes become less responsive to oxidative stress. A) Western blotting of whole cell lysates from fresh cultures (passaged up to three times) had consistent increases in GFAP, PGC-1α and HSP70, and a decrease in GS, in response to increasing concentrations of PQ (as described in Figure 3). B) Cells that had been subcultured (passaged more than three times) tended to lose their responsiveness to PQ, such that there was little change in the same marker panel. Therefore fresh cultures were used for the subsequent experiments in this report. (TIF) [file pone.0083049.s002.tif]
